# A unique case of spontaneous regression of metastatic papillary renal cell carcinoma: a case report

**DOI:** 10.4076/1757-1626-2-7769

**Published:** 2009-08-11

**Authors:** Rebecca Lim, Puay Hoon Tan, Christopher Cheng, Thirugnanam Agasthian, Hwei Ling Tan, Bin Tean Teh, Min-Han Tan

**Affiliations:** 1 Department of Medical Oncology, National Cancer Centre SingaporeSingapore 169610Republic of Singapore; 2 Department of Pathology, Singapore General Hospital, SingaporeSingapore 169608Republic of Singapore; 3 Department of Urology, Singapore General Hospital, SingaporeSingapore 169608Republic of Singapore; 4 Department of Cardiothoracic Surgery, National Heart CentreSingapore 168752Republic of Singapore; 5 NCCS-VARI Laboratory of Translational Cancer Research, SingaporeSingapore 169610Republic of Singapore

## Abstract

Spontaneous regression of cancer is a rare, but well documented, phenomenon. We present a unique case of an 82 year old Chinese male who experienced spontaneous regression of histologically-verified metastatic type II papillary renal cell carcinoma in the absence of intervening systemic therapy or surgery. This is the first reported case of spontaneous regression of papillary renal cell carcinoma. The mechanism of spontaneous regression remains unknown, and represents a challenge for existing oncology paradigms.

## Introduction

Renal cell carcinoma (RCC) accounts for 3% of all new cancer cases worldwide with a rising incidence [1]. The first reported case of spontaneous regression of metastatic RCC was published in 1928 [2]. Since then, multiple case reports and small series have highlighted this rare but definite phenomenon. The frequency of this phenomenon is estimated to be <1%. Further limiting available data, most reported instances of spontaneously regressed metastases do not have histologic confirmation. In most reports, the regression occurs shortly after a nephrectomy [3]. Almost all documented cases of spontaneous regression of RCC are reported to occur in the metastasis rather than the primary lesion. Herein, we present a cytologically verified case of lung metastasis following relapse from papillary RCC, which regressed spontaneously over twelve months without therapy.

## Case presentation

**Figure 1.**
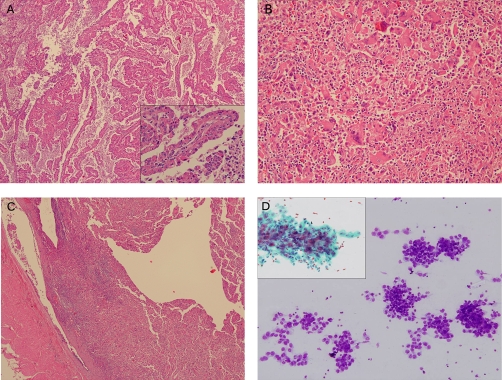
**(A)** Papillary RCC composed of fibrovascular fronds covered by cells with pink cytoplasm. Inset shows high magnification of the cells lining the fronds, revealing cytologic nuclear atypia and oncocytic cytoplasm (type II features). **(B)** Sarcomatoid component of the RCC, with loosely aggregated epithelioid and spindled pleomorphic cells with occasional multinucleation. Accompanying inflammatory cells are present. **(C)** Tumour thrombus within the renal vein. **(D)** Fine needle aspiration cytology of the lung lesion, revealing morular, loosely cohesive clusters of abnormal epithelial cells (Diff-Quik). Inset shows a PAP stained finger-like conglomerate of cells with enlarged vesicular nuclei and prominent nucleoli, in keeping with metastatic papillary RCC.

**Figure 2.**
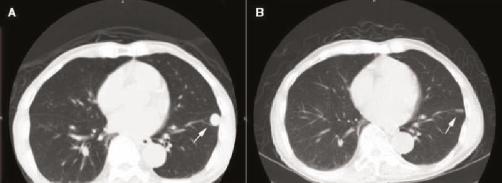
**(A)** The pulmonary metastasis in the left lower zone of the lung 6 years post-nephrectomy. **(B)** A CT scan one year post relapse showing near complete regression of the lesion.

A 75 year-old Singaporean male of ethnic Chinese origin was diagnosed with localized right-sided RCC following a work-up for an elevated creatinine level (134 umol/L) discovered on routine health screening. Computed tomographic (CT) scanning of the lungs did not reveal any metastases. A radical nephrectomy without lymph node dissection was performed, and histology revealed a high-grade Type II papillary RCC (Figure 1A), with sarcomatoid change (Figure 1B). Tumor invasion of the renal vein and renal pelvis was noted (Figure 1C) with a final stage of IIIB. The patient was placed on surveillance with regular chest X-rays for the first 3 years of follow-up, with yearly visits thereafter. 6 years following surgery, CT scanning revealed a single solid pulmonary lesion in the left lower lobe of the lung (Figure 2A) with no other sites of disease. A percutaneous biopsy of this lesion yielded papillary RCC (Figure 1D). Wedge resection of the nodule was offered, but was declined by the patient. No systemic therapy was offered to the patient as there was no established standard of care for metastatic non-clear cell RCC at that point in time, and he did not consume any new drugs or supplements. Over the next 12 months, imaging revealed spontaneous regression of the lesion (Figure 2B). He remains free of progressive disease 24 months after relapse.

## Discussion

Since the first observation of spontaneous regression in 1928 [2], there has been little or no advance in our understanding of this unusual phenomenon in cancer. The actual incidence of spontaneous regression of kidney cancer is poorly understood, as there is little information on the true denominator of patients with RCC. While spontaneous regression of clear cell RCC is recognized as a rare phenomenon, spontaneous regression of papillary RCC has not been previously reported. Our institutional database of RCC has a series of 80 patients with papillary RCC over approximately 20 years, none of whom exhibited spontaneous regression.

The mechanism for spontaneous regression of metastasis from RCC is unknown. The favored hypothesis to date is that spontaneous regression occurs due to immunological factors, including the removal of a prometastatic or growth factor secreted by the tumour resulting in apoptosis [4]. In observed regressions of RCC, regressions have occurred following plasma infusion from patients who have experienced a regression, suggesting that humoral factors may play a role [5]. Cytokines, namely interferon and interleukin 2 exert anti-tumour affects by for example, inhibiting angiogenesis of the tumour [5].

It has been speculated that resection of the primary tumour may result in the removal of a systemic stimulatory growth factor and thus directly result in regression [4]. Our case report demonstrating progression and regression of relapsed tumor many years after nephrectomy suggests that this mechanism may not be responsible for all cases of regression.

To date, few reports of spontaneous regression of RCC have pathological documentation, and even fewer document the specific subtype. A review we undertook of these case reports indicate either a clear cell subtype or do not specify the subtype (references available on request). The issue of subtyping in RCC is particularly important. Metastatic clear cell RCC may occasionally undergo a durable complete remission following high dose interleukin-2 therapy, but this is not recognized in non-clear cell RCC including papillary RCC, supporting an immunologic mechanism in spontaneous regression of clear cell RCC [6]. Our case report represents the first such spontaneous regression observed in a patient with papillary RCC. Homozygous inactivation of the von Hippel-Lindau (*VHL*) gene is a common molecular abnormality in both sporadic and familial forms of clear cell carcinomas, with a resulting dysregulation of the hypoxia response pathway, increased angiogenesis and tumour growth [7]. Underlying pathways driving papillary RCC growth are less well established. Metabolic signaling has been implicated through the identification of a familial cancer syndrome including type 2 papillary RCC from an underlying germline mutation in the fumarate hydratase (FH) gene, and different activation patterns of cell cycle pathways between type I and type II papillary RCC have been reported [8]. Both clear cell RCC and papillary RCC are thought to be of tubular origin [9].

Sarcomatoid variants of RCC are biologically very aggressive tumours that respond poorly to immunotherapy [10]. It is thought that sarcomatoid change represents an active area of epithelial- mesenchymal transition. Our patient had a high grade papillary RCC with sarcomatoid change and renal vein invasion relapsing only 6 years later, which was quite unexpected. Whether this long disease-free interval is directly relevant to spontaneous regression is a matter for speculation.

When the patient was evaluated initially at relapse, there was no standard of care for systemic therapy for metastatic non-clear cell RCC, and he was placed on observation. In recent years, targeted therapies in this patient group have been studied with some success, these agents including temsirolimus [11], sunitinib and sorafenib [12].

In summary, we report the first case of spontaneous regression in a patient with relapsed metastatic papillary RCC undergoing surveillance.

## Abbreviations

CT: computed tomographic
FH: fumarate hydratase
RCC: renal cell carcinoma
VHL: von Hippel-Lindau
